# Evolution characteristics of dynamic balance disorder over the course of PD and relationship with dopamine depletion

**DOI:** 10.3389/fnagi.2022.1075572

**Published:** 2023-02-02

**Authors:** Jing Gan, Xiaodong Wu, Ying Wan, Jiahao Zhao, Lu Song, Na Wu, Hui Wang, Yafu Yin, Zhenguo Liu

**Affiliations:** ^1^Department of Neurology, Xinhua Hospital Affiliated to Shanghai JiaoTong University School of Medicine, Shanghai, China; ^2^Department of Nuclear Medicine, Xinhua Hospital Affiliated to Shanghai Jiao Tong University School of Medicine, Shanghai, China

**Keywords:** Parkinson's disease, dynamic balance, dopamine transporter (DAT), dopaminergic depletion, white matter hyperintensities (WMHs)

## Abstract

**Objective:**

This study aimed to assess the evolution of dynamic balance impairment during the course of Parkinson's disease (PD) and to clarify the contribution of striatal dopaminergic innervation to poor dynamic balance.

**Methods:**

In our study, 89 patients with PD (divided into 2 groups according to the H-Y stage) and 39 controls were included. Kinematic data were recorded by a portable inertial measurement unit system. Dopaminergic loss in the striatal subregion was verified through the ^11^C-CFT PET examination. The severity of white matter hyperintensities (WMHs) was assessed by the Scheltens scale. The correlation between dynamic kinematic parameters and dopamine transporter availability was analyzed by multivariate regression analysis.

**Results:**

Patients with early PD presented with imbalance featured by smaller three-dimensional trunk ROM with reduced trunk coronal angular velocity during walking and with reduced trunk sagittal angular velocity during the stand-to-sit task (all *p* < 0.05). These abnormalities were not more severe at a later stage. The ROM in the coronal and transverse planes during walking correlated with caudate DAT uptake (β = 0.832, *p* = 0.006, *Q* = 0.030, and β = 0.890, *p* = 0.003, *Q* = 0.030) after controlling for age, gender, and WMHs. As the disease progressed, the trunk sagittal and transverse angular velocities during walking and trunk sagittal angular velocity when turning and sitting-to-standing were slower, which was accompanied by reduced gait velocity gradually (all *p* < 0.05). These parameters related to disease progression have no association with striatal DAT uptake (all *p* > 0.05).

**Conclusion:**

The dynamic balance in PD was impaired from the early stages, and the characteristics of the impairment changed differently as the disease progressed. Dopaminergic denervation has a lower contribution to dynamic balance disorders throughout PD.

## Introduction

Parkinson's disease (PD) is a progressive neurodegenerative disease, with an increasing incidence in elderly individuals over the age of 60 (Zhang et al., 2005). The cardinal symptoms of PD are bradykinesia, rigidity, tremor, and instability. The imbalance or instability of patients with PD occurs predominately during dynamic movements, such as walking, turning, or postural transitions, which was considered dynamic balance impairment (Mellone et al., 2016; Kuhman et al., 2020; Sloot et al., 2020). Dynamic balance refers to the ability to control the center of mass (COM) of the body over its moving base of support or to stabilize COM within a series of alternating unilateral stances (Mellone et al., 2016; Siragy and Nantel, 2018; Kuhman et al., 2020). Poor dynamic balance contributes to an increase in the risk of falls and immobility in patients with PD. Clinicians often analyzed the scores of the motor performance tests (e.g., Berg scale, Timed Up and Go, etc.) to assess dynamic balance for patients with PD (Hubble et al., 2015). Usually, the evaluation scores of these tests were always normal in the early stage of PD, although some patients with PD in the early stage expressed a sense of balance instability clinically (Zampieri et al., 2010; Zampogna et al., 2021). The emergence of postural instability or dynamic imbalance was considered as the transition from Hoehn and Yahr (H-Y) stage 2 to stage 3, which marked a pivotal milestone in PD (Bohnen et al., 2011). Most studies on dynamic balance disorders of PD focused on patients with moderate-advanced disease and fallers.

At present, it is unclear if dynamic balance affects patients with early PD and how dynamic balance impairment evolves with the progression of the disease. Previous research showed that the Timed Up and Go test was not sensitive to subtle abnormalities present in patients with early PD when balance and gait problems are not clinically evident (Zampieri et al., 2010). With the development of motor analysis technology, evaluation of the dynamic balance could be objectively quantified, and this could provide information on stability impairment in patients with PD.

The loss of dopaminergic neurons causes impairments in the function of the basal ganglia, which results in motor impairments in patients with PD. The dopamine depletion begins in the substantia nigra pars compacta, propagating further into additional structures of the basal ganglia (Poewe et al., 2017). The striatal dopamine reduction was positively correlated with the severity of rigidity and bradykinesia in PD, which was indicated by dopamine transporter positron emission tomography (DAT-PET) (Kaasinen and Vahlberg, 2017; Kuribara et al., 2020). Stability and balance during the movement process involve many abilities. Except for the execution of the automatic movements modulated by striatal dopaminergic neurons, the inter-limb coordination, the adaptive neuromuscular system, and locomotor patterns requiring frontal lobe cognitive and executive functions were also involved in the dynamic balance (Mellone et al., 2016). In addition, the evidence demonstrated that comorbid white matter hyperintensities (WMHs) negatively influenced the severity of balance and gait in patients with PD (Bohnen et al., 2011; Craig et al., 2019; Lee et al., 2020). PD is a dysfunction of the multi-neurotransmitter systems; the contribution of dopamine loss in the striatum to dynamic balance in patients with PD is unclear. However, this information is important to determine the strategy of medical therapy.

In this study, we applied the wearable system with collection parameters in real-time to evaluate the gait and dynamic balance of patients with PD ranging from H-Y stage 1 to stage 4. Meanwhile, the dopamine depletion of the patients was assessed by DAT-PET and their WMHs by cerebral MRI. Our objectives were to (1) determine the characteristics of dynamic balance in the early to moderate stage of PD and how dynamic balance impairments evolve with the progression of the disease and (2) clarify the contribution of striatal dopaminergic innervation to dynamic balance impairment in PD.

## Methods

### Participants

In this study, 89 idiopathic patients with PD and 39 healthy older controls were enrolled between January 2019 and September 2022. All patients were recruited from the Department of Neurology at Xinhua Hospital, Shanghai Jiao Tong University School of Medicine. The inclusion criteria of the PD group were (1) diagnosis in terms of the MDS Clinical Diagnostic Criteria for Parkinson's disease (Postuma et al., 2015); and (2) no history of orthopedic or musculoskeletal disorders and no other causes (e.g., ophthalmic disorder) that could impair the balance or gait. The exclusion criteria of the PD group were (1) the Hoehn-Yahr (H-Y) stage of 5; (2) an Mini-Mental State Examination (MMSE) score of <24; and (3) patients who had undergone deep brain stimulation or had severe cardiovascular, pulmonary, digestive, and psychiatric diseases. The parkinsonian-related information (e.g., age, gender, height, and disease duration) was collected. The motor and cognitive assessment scales included the MDS-Unified Parkinson's Disease Rating Scale (MDS-UPDRS), the Hoehn-Yahr (H-Y) stage, the MMSE score, and the Frontal Assessment Battery (FAB). Information on pharmacological treatment was collected and calculated in total daily levodopa equivalent dose (LED) (Tomlinson et al., 2010). All patients underwent dopamine transporter (DAT) imaging using ^11^C-2β-carbomethoxy-3β-(4-fluorophenyl) tropane (^11^C-CFT) PET scans and showed appropriate DAT defects in the posterior putamen on CFT PET scans (Oh et al., 2012). The patients received 3.0T magnetic resonance imaging (MRI) scanning of the brain if the contraindications were not found.

The healthy controls were partners of patients with PD or volunteers. They were excluded if they reported previously having neurological diseases, orthopedic disorders, or musculoskeletal disorders that could impact balance or gait. The study was approved by the Research Ethics Committee of Xinhua Hospital, affiliated with Jiao Tong University, School of Medicine. All participants gave their signed, informed consent. Finally, 39 controls (16 men and 23 women) were recruited in the study (mean age: 65.74 ± 10.13 years) and 89 patients with PD (45 men and 44 women) were in the PD group (mean age: 67.02 ± 9.21 years). The age of the PD diagnosis was 63.87 ± 9.62 years.

### Kinematic analysis of gait and dynamic balance

**Figure 1:**
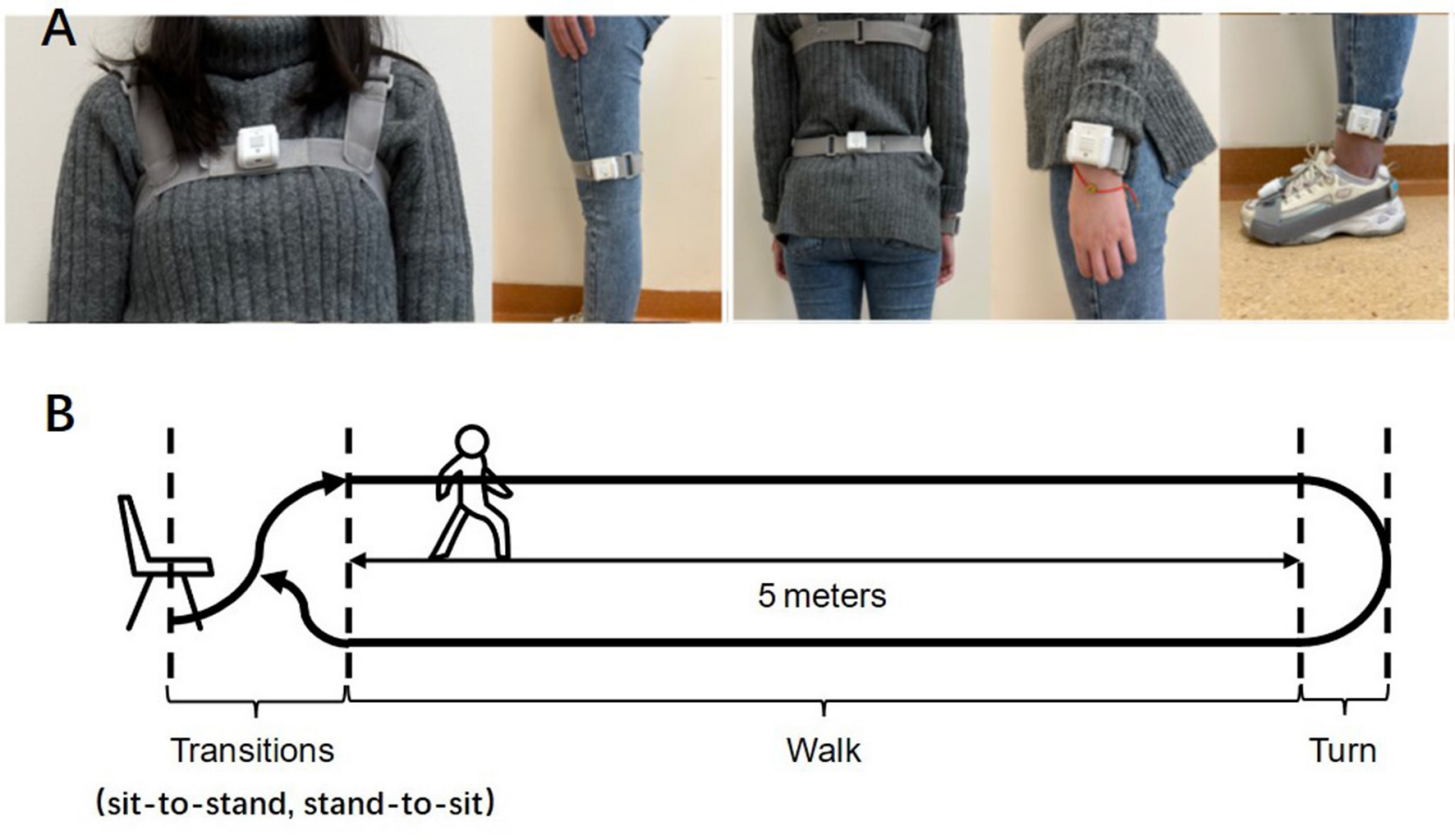
**(A, B)** The front, back and side view of wearable device and the process of motor task.

The wearable inertial measurement unit (IMU) system (GYENNO Science, Shenzhen, China) was applied in our study to collect kinematic information with sensors in real-time (Zago et al., 2018; Cao et al., 2020). Participants wore 10 inertial sensors placed bilaterally on the lower back, the chest (sternum), and the bilateral feet, ankles, thighs, and wrists by elastic belts (Figure 1A). The participants were asked to do the following motor tasks: (1) stand up from the chair, (2) walk straight for a 5-m distance, (3) turn and return to the start point, and (4) sit down (Figure 1B). After the investigator confirmed that each sensor was placed correctly, the participant sat on the chair at the start point. When the investigator issued the instruction, they began to stand, walk straight, and then turn, walk back, and sit down.

The kinematic data were transmitted to the host computer *via* a Bluetooth link for further processing and storage. The trunk range of motion (ROM) and trunk peak angular velocity of participants in the coronal, sagittal, and transverse planes were calculated during the walking condition. The following gait parameters were extracted when walking straight: gait velocity, cadence, step length, and stride length. Turning duration and trunk sagittal peak angular velocity were calculated when turning. Trunk sagittal peak angular velocity, trunk sagittal ROM, and average duration were calculated when the position changed (sit-stand shift). Trunk ROM and peak velocity in three planes of motion were often used to detect dynamic postural balance and to show the displacement of trunk sway, which was considered an assessment of axial impairment and imbalance in patients with PD (Jehu and Nantel, 2018; Cano-de-la-Cuerda et al., 2020). The values for all variables were averaged for statistical analysis. Patients with PD were evaluated during the “OFF” period (assessment was done 12 h after anti-PD drug withdrawal).

### PET-CT with ^11^C-2β-carbomethoxy-3β-(4-fluorophenyl) tropane (^11^C-CFT)

PET-CT examinations were performed at least 12 h after the patient stopped using dopaminergic drugs. The scans were performed on a Biograph 64 system (Siemens Healthineers, Erlangen, Germany) with a 21.6-cm axial field of view. The patient rested quietly for approximately 30 min before the examination, and 370 MBq of the tracer ^11^C-CFT was injected through the elbow vein (synthesized by the cyclotron of Xinhua Hospital, radiochemical purity >90%). The patient was then required to stay calm in a quiet, dark environment and allowed to take rest for 60 min. Brain imaging was then initiated. PET image acquisition, which took approximately 15–20 min, was done in 3D mode. Using an iterative reconstruction algorithm with a layer thickness of 5 mm, the images were attenuated and corrected by CT to obtain PET-CT images of the transverse, sagittal, and coronal planes, and their positions were aligned to the standard anatomical positions.

On the PET-CT fusion imaging, three consecutive layers of the clearest caudate nucleus and putamen were selected, and the regions of interest (ROI) of the bilateral caudate nucleus, the anterior putamen, and the posterior putamen were manually delineated layer by layer. The cerebellum lacking DAT distribution was taken as the reference region, and the semiquantitative value of DAT distribution in each ROI region was obtained according to the formula, which is defined as DAT binding: DAT binding = (caudate or putamen radioactivity count/cerebellar radioactivity count) – 1. The DAT binding values of the caudate nucleus, the anterior putamen, and the posterior putamen are the average DAT binding values of the left and right sides.

### MRI evaluation

Magnetic resonance imaging was conducted using a 3.0 T scanner (GE Signa). In accordance with our previous study (Wan et al., 2019), the imaging protocol involved T1-weighted imaging [repetition time (TR) 2,200 ms, echo time (TE) 24 ms, and section thickness 5 mm] and T2-weighted fluid-attenuated inversion recovery (T2 FLAIR, TR 8,500 ms, TE 120 ms, and section thickness 5 mm). Structural MRI was analyzed by an experienced observer who was blind to the clinical data. It was characterized by a hypointense lesion and hyperintense rim on FLAIR images, according to the corresponding hyperintensity and hypointensity on T2 and T1 images, respectively. WMHs were defined as hyperintense lesions on FLAIR images. The visual rating scale of WMHs was assessed by two neurologists separately using the Scheltens scale in a semiquantitative manner (Scheltens et al., 1993; Wan et al., 2019; Jeong et al., 2021).

### Statistical analysis

The data of our study were presented as mean value ± standard deviation (SD). The categorical statistics were recorded as count data. ANOVA with *post-hoc* comparisons was performed for group differences across the PD groups and the control group. The chi-squared test or *t*-test was used to analyze group differences between PD group 1 and PD group 2 (including disease duration, MDS-UPDRS III, MMSE, FAB, and LED). Spearman's correlation tests were used to correlate the MDS-UPDRS III score and DAT availability of the posterior putamen. The multivariate linear regression analyses were performed to identify the effects of striatal DAT availability on dynamic balance control in PD. The kinematic parameters which were different from controls and the kinematic parameters which were related to disease progression were analyzed separately. The results with a *p*-value of < 0.05 were considered statistically significant. The false discovery rate (FDR) method was used to reduce the type I error for multiple comparisons. We also presented *Q* values that were FDR-corrected *p*-values for multiple comparisons. The results with *Q* of < 0.05 were regarded as statistically significant, and the results with 0.05 ≤ *Q* < 0.1 were considered a trend toward significance to increase the statistical power. The data were analyzed using SPSS 22.0.

## Results

### The differences in clinical, gait, and balance measures among patients with early PD, patients with mild-moderate PD, and healthy controls

The demographic and clinical characteristics of all participants are shown in Table 1. According to the H-Y stage, patients with PD were classified into PD group 1 (H-Y ≤ 2) and PD group 2 (H-Y > 2). There was no difference in sex distribution and height among PD group 1, PD group 2, and the control group (both *p* >0.05). The patients in PD group 2 were significantly older than those in the control group (71.58 ± 6.39 vs. 65.74 ± 10.13, *p* = 0.009) or those in PD group 1 (71.58 ± 6.39 vs. 64.54 ± 9.60, *p* = 0.001), while there was no difference between the control group and PD group 1 (*p* = 0.527). Concerning patients with PD, the patients in PD group 2 presented a longer disease duration (3.87 ± 3.43 vs. 2.77 ± 1.51, *p* = 0.038), higher MDS-UPDRS III scores (36.33 ± 13.19 vs. 20.65 ± 12.89, *p* = 0.000), and took more antiparkinsonian medication (366.38 ± 321.43 vs. 145.09 ± 169.64, *p* = 0.001) than those in PD group 1. However, no statistical difference in MMSE and FAB scores was found between PD group 1 and PD group 2 (*p* = 0.614 and *p* = 0.070, respectively).

**Table 1:** Demographic information of two PD groups and control group.

	**Control group**	**PD group 1 (H-Y ≤ 2)**	**PD group 2 (H-Y > 2)**	**Statistical value**	***p*-value**	**p1 value**	**p2 value**	**p3 value**
	**(*****n** =* **39)**	**(*****n** =* **58)**	**(*****n** =* **31)**					
Sex (male/female)	16/23	29/29	16/15	1.010 ^a^	0.604	/	/	/
Age (years)	65.74 ± 10.13	64.54 ± 9.60	71.58 ± 6.39	6.264 ^b^	0.003	0.527	0.009	0.001
Height (cm)	163.87 ± 7.79	165.63 ± 8.21	164.29 ± 9.39	0.575 ^b^	0.564	0.315	0.836	0.475
Disease durations (years)	/	2.77 ± 1.51	3.87 ± 3.43	−2.105 ^c^	0.038	/	/	/
MDS-UPDRS III scores	/	20.65 ± 12.89	36.33 ± 13.19	−5.202 ^c^	0.000	/	/	/
MMSE scores	/	27.21 ± 3.17	26.87 ± 2.66	0.507 ^c^	0.614	/	/	/
FAB scores	/	15.37 ± 3.03	13.93 ± 3.80	1.840 ^c^	0.070	/	/	/
LED (mg/day)	/	145.09 ± 169.64	366.38 ± 321.43	−3.518 ^c^	0.001	/	/	/

^a^Data were performed for group differences with the chi-squared test. ^b^Data were performed for group differences with ANOVA. P1, *post-hoc* comparisons p-value of control group vs. PD group 1; P2, *post-hoc* comparisons p-value of control group vs. PD group 2; P3, *post-hoc* comparisons p-value of PD group 1 vs. PD group 2. ^c^Data were performed for 2 group differences with t-test.

Table 2 shows the comparison of the kinematic parameters of gait and dynamic stability among the three groups. There were statistical differences in gait velocity, step length, and stride length among these three groups (*p* < 0.05). The gait velocity gradually slowed down in the three groups (*F* = 40.21, *p* = 0.000). The fastest gait velocity among the three groups was in the control group (1.04 ± 0.20 m/s), followed by that in the PD group 1 (0.95 ± 0.19 m/s, *p* = 0.031), and the slowest was that in the PD group 2 (0.64 ± 0.20 m/s, *p* = 0.000). The step length in PD group 2 was the shortest among the 3 groups (37.21 ± 11.43 cm), which was significantly shorter than that in the control group (56.07 ± 9.80 cm, *p* = 0.000) and PD group 1 (52.91 ± 8.51 cm, *p* = 0.000). However, no difference was found between the PD group 1 and the control group (*p* = 0.121). Similarly, the stride length in the PD group 2 (73.56 ± 22.29 cm) was shorter than that in the control group (111.37 ± 19.44 cm, *p* = 0.000) and the PD group 1 (105.23 ± 16.80 cm, *p* = 0.000), but there was no difference between that in the control group and the PD group 1 (*p* = 0.127). There was no difference in cadence among the three groups (*F* = 2.198, *p* = 0.115).

**Table 2:** Kinematic parameters of two PD groups and control group.

		**Control group**	**PD group 1 (H-Y ≤ 2)**	**PD group 2 (H-Y > 2)**	**Statistical value**	***p*-value**	**p1 value**	**p2 value**	**p3 value**
		**(*****n** =* **39)**	**(*****n** =* **58)**	**(*****n** =* **31)**					
Gait velocity (m/s)		1.04 ± 0.20	0.95 ± 0.19	0.64 ± 0.20	40.21	0.000	0.031	0.000	0.000
Candence (step/min)		111.84 ± 7.55	109.40 ± 13.30	105.40 ± 16.24	2.198	0.115	0.363	0.039	0.162
Step length (cm)		56.07 ± 9.80	52.91 ± 8.51	37.21 ± 11.43	37.108	0.000	0.121	0.000	0.000
Stride length (cm)		111.37 ± 19.44	105.23 ± 16.80	73.56 ± 22.29	38.63	0.000	0.127	0.000	0.000
Turning	Average duration (sec)	1.43 ± 0.06	1.86 ± 0.79	3.16 ± 1.88	24.308	0.000	0.059	0.000	0.000
	Trunk sagittal peak angular velocity (degree/sec)	174.85 ± 25.46	135.13 ± 27.27	94.78 ± 25.98	79.73	0.000	0.000	0.000	0.000
Walking									
Coronal	Trunk coronal peak angular velocity (degree/sec)	24.53 ± 6.09	21.40 ± 6.32	20.28 ± 6.63	4.423	0.014	0.020	0.006	0.426
	Trunk ROM Roll (degree)	5.41 ± 2.29	3.24 ± 1.31	3.68 ± 1.60	18.726	0.000	0.000	0.000	0.259
Sagittal	Trunk sagittal peak angular velocity (degree/sec)	38.82 ± 11.73	33.67 ± 12.28	25.33 ± 6.66	12.968	0.000	0.027	0.000	0.001
	Trunk ROM Pitch (degree)	5.22 ± 1.45	4.27 ± 1.40	3.84 ± 0.89	10.444	0.000	0.001	0.000	0.149
Transverse	Trunk transverse Peak Angular Velocity (degree/sec)	46.03 ± 13.01	41.27 ± 11.65	34.64 ± 10.42	7.977	0.001	0.057	0.000	0.013
	Trunk ROM Yaw (degree)	11.14 ± 3.37	9.15 ± 3.06	9.57 ± 3.45	4.408	0.014	0.004	0.049	0.563
Sit to stand	Average duration (sec)	1.43 ± 0.49	1.73 ± 0.74	2.69 ± 1.28	20.206	0.000	0.098	0.000	0.000
	Trunk sagittal peak angular velocity (degree/sec)	87.04 ± 22.73	68.84 ± 19.36	53.01 ± 18.92	23.849	0.000	0.000	0.000	0.001
	Trunk sagittal ROM (degree)	37.36 ± 12.12	34.48 ± 7.18	35.17 ± 10.48	1.025	0.362	0.160	0.359	0.753
Stand to sit	Average duration (sec)	1.91 ± 0.46	2.19 ± 0.84	2.84 ± 1.00	12.121	0.000	0.101	0.000	0.000
	Trunk sagittal peak angular velocity (degree/sec)	83.06 ± 20.20	64.48 ± 20.53	60.73 ± 20.64	12.791	0.000	0.000	0.000	0.419
	Trunk sagittal ROM (degree)	38.02 ± 9.56	33.51 ± 10.51	34.29 ± 8.74	2.515	0.085	0.031	0.123	0.726

Data were performed for group differences with ANOVA. P1, *post-hoc* comparisons p-value of control group vs. PD group 1; P2, *post-hoc* comparisons p-value of control group vs. PD group 2; P3, *post-hoc* comparisons p-value of PD group 1 vs. PD group 2.

When walking, there were significant differences among the three groups in the trunk ROM in the coronal, sagittal, and horizontal planes (*p* = 0.000, 0.000, and 0.014, respectively). The largest trunk ROM of the coronal, sagittal, and horizontal axes among the three groups was in the control group (5.41 ± 2.29, 5.22 ± 1.45, and 11.14 ± 3.37, respectively), which were significantly larger than those in PD group 1 (3.24 ± 1.31, 4.27 ± 1.40, and 9.15 ± 3.06, *p* = 0.000, 0.001, and 0.004, respectively) and those in PD group 2 (3.68 ± 1.60, 3.84 ± 0.89, and 9.57 ± 3.45, *p* = 0.000, 0.000, and 0.049, respectively). However, there was no difference in trunk ROM in the 3 axes between PD group 1 and PD group 2 (*p* > 0.05). The duration of turning, sitting-to-standing, and standing-to-sitting were gradually prolonged in the control group, the PD group 1, and the PD group 2. There were statistical differences in all durations among these three groups (*p* < 0.000). However, there was no difference in these durations between the control group and the PD group 1 (*p* = 0.059, 0.098, and 0.101, respectively). The trunk sagittal peak angular velocity during walking, turning, sitting-to-standing, and standing-to-sitting was progressively slower among these three groups (*p* < 0.000). There was no difference in the trunk sagittal ROM during sit/stand position change (sitting-to-standing *F* = 1.025, *p* = 0.362; stand-to-sit *F* = 2.515, *p* = 0.085).

These data suggested that, in the early stage of PD (H-Y ≤ 2), the ROM on the coronal, sagittal, and transverse axes during walking was significantly reduced compared with the control group, but these differences did not worsen compared with patients with PD in the H-Y > 2 stage. Other parameters, such as the gait velocity, the trunk peak angular velocity during walking, and the sit-stand change, became lower in patients with early PD and more serious in patients with mild-moderate PD. The rest of the parameters, such as step length and stride length, were significantly shorter in patients with mild-moderate PD but not in patients with early PD compared to the control.

### Association between dynamic balance parameters and dopamine depletion

Furthermore, we analyzed the relationship between dopamine depletion and kinematic parameters in patients with PD. The ^11^C-CFT uptake value was 1.78 ± 0.85 in the caudate and 2.44 ± 1.44 in the putamen (1.50 ± 0.80 in the anterior putamen and 0.94 ± 0.70 in the posterior putamen). DAT availability in the posterior putamen was well correlated with a negative MDS-UPDRS III score (r_s_ = −0.333, *p* = 0.004). To avoid the effect of comorbid WMH in the brain on gait and balance, we used the Scheltens scale in statistics to adjust. Cerebral MRI was performed in 62 of the 89 patients with PD. Among them, 6 patients with PD do not have comorbid WMHs. The score of total WMH in the PD group was 12.11 ± 9.19, ranging from 0 to 46.

In this part, we first chose the kinematic parameters that differed only between the control group and the PD group 1. This suggested that these parameters did not correlate with disease progression. We used them as a dependent variable to determine the impact of striatal dopaminergic depletion on dynamic imbalance. Linear regression analysis was performed, and age, gender, and total WMH scores were adjusted as covariates (shown in Table 3). After controlling for age, gender, and white matter hyperintensities, the regression models using trunk coronal ROM as a dependent variable revealed that trunk coronal ROM had a significant positive correlation with caudate uptake (β = 0.832, *p* = 0.006, and *Q* = 0.030). Regression models using the trunk transverse ROM as a dependent variable showed that it was also positively associated with caudate uptake (β = 0.890, *p* = 0.003, and *Q* = 0.030). It had no significant correlation with the caudate and putamen uptake when the dependent variables were trunk coronal angular velocity, trunk sagittal ROM during walking, or trunk sagittal angular velocity when standing-to-sitting.

**Table 3:** Multivariate linear regression analyses for the DAT uptake in each striatal subregion for parameters different from controls.

**Striatal subregion/dynamic kinematic parameters**	**Uptake**					
	**Caudate**			**Putamen**		
	β **(95% CI)**	***P***	***Q***	β **(95% CI)**	***P***	***Q***
**Walking**						
Trunk coronal Peak Angular Velocity	0.364 (-2.134-8.394)	0.238	0.340	−0.429 (−5.093-0.837)	0.156	0.312
Trunk coronal ROM	0.832 (0.496-2.751)	0.006	0.030	−0.550 (−1.254- 0.071)	0.056	0.187
Trunk sagittal ROM	0.155 (-0.705-1.208)	0.599	0.666	0.024 (−0.517-0.561)	0.935	0.935
Trunk transverse ROM	0.890 (1.334-6.328)	0.003	0.030	−0.545 (−2.758-0.055)	0.059	0.148
**Stand to sit**						
Trunk Sagittal Peak Angular Velocity	0.352 (-7.382-25.830)	0.270	0.338	−0.380 (−15.061-3.612)	0.224	0.373

As previously mentioned, the gait velocity, the trunk sagittal peak angular velocity during walking, turning, and sit-to-stand change, and the trunk transverse peak angular velocity during walking gradually slowed down with H-Y stage aggravation, indicating that these parameters were related to disease progression. Thus, we chose these kinematic parameters that were associated with disease progression for linear regression analysis to determine the impact of striatal DAT availability on the dynamic balance after adjusting for age, gender, disease duration, MDS-UPDRS III, gait velocity, and the total WMH score. Table 4 demonstrates the results of multivariate linear regression analysis. There was no association between these dynamic balance parameters and DAT uptake of the caudate or the putamen.

**Table 4:** Multivariate linear regression analyses for the DAT uptake in each striatal subregion for parameters related to disease progression.

**Striatal subregion/dynamic kinematic parameters**	**Uptake**					
	**Caudate**			**Putamen**		
	β **(95% CI)**	***P***	***Q***	β **(95% CI)**	***P***	***Q***
**Turning**						
Turning peak angular velocity	−0.007 (−16.709-16.144)	0.972	1.111	0.247 (−4.092-15.473)	0.247	1.976
**Walking**						
Trunk sagittal peak angular velocity	0.114 (−4.173-6.828)	0.629	1.006	−0.006 (−3.317-3.234)	0.980	0.980
Trunk transverse Peak Angular Velocity	0.311 (−3.252-12.156)	0.250	1.000	−0.222 (−6.402-2.774)	0.429	1.144
**Sit to stand**						
Trunk sagittal peak angular velocity	0.122 (−11.735-18.394)	0.658	0.877	−0.208 (−12.121-5.699)	0.471	0.942

## Discussion

Our study showed the evolution characteristics of dynamic balance disorders during the course of PD and its association with striatal dopamine depletion. The dynamic balance was impaired in the early stage of PD, with different aspects and characteristics of damage during the disease progression. The smaller three-dimensional trunk ROM during walking and reduced trunk sagittal angular velocity when standing-to-sitting appeared at the early stage of PD and did not aggravate disease progression. The walking ROM in the coronal and transverse planes is positively correlated with decreased DAT availability in the caudate. In addition, the gait speed and the trunk sagittal peak angular velocity during walking, turning, and sit-to-stand change gradually slowed down with H-Y stage aggravation and did not correlate with dopamine depletion.

Deficits in balance and stability are common and disabling features of PD. Many clinical assessments may not be sufficiently sensitive to detect changes in balance during walking, turning, or position transition in patients with PD who have mild to moderate disease severity. There are many methods to quantify dynamic balance, and each method reflects distinct information about stability impairment. However, there has been no consensus on the assessment methods for PD (Hubble et al., 2015; Siragy and Nantel, 2018). A recent meta-analysis indicated that wearable sensors may be useful for evaluating balance and walking stability in individuals with PD, but no recommendations were made for the most appropriate placements and outcomes of wearable sensors (Hubble et al., 2015; Siragy and Nantel, 2018). In our study, we used a wearable sensor to quantify the Timed Up and Go test and obtain the range of motion of the trunk and the angular velocity of the trunk when the base of support changed (Visser et al., 2007; Cao et al., 2020). The walking distance extended from 3 m to 5 m.

### Dynamic imbalance evolution at distinct stages

According to our findings, most variables of balance and gait were affected in the more advanced stages of PD (Zampieri et al., 2010; Mellone et al., 2016; Jehu and Nantel, 2018; Mirelman et al., 2019). Patients with PD in our study showed a progressive reduction in trunk angular velocity when motion, turning, and walking, with a gradually reduced gait speed and shortened step length and stride length. Such deficits worsened with the severity of the disease from H-Y stages 1–2 to H-Y stages 3–4, indicating that partially dynamic balance control and gait gradually worsened (Jehu and Nantel, 2018). However, except for walking coronal angular velocity and sagittal trunk angular velocity when standing-to-sitting, there was no statistical significance in these parameters between patients with PD at the early stage and controls, indicating that these parameters might be more useful for assessing disease progression than for early diagnosis. Slower speed and reduced angular velocity when turning or changing positions in PD were considered potential compensatory strategies to prevent dynamic postural instability (Mellone et al., 2016). These deficits were consistent with “en bloc” trunk motion in PD and partially in line with the previous findings (Visser et al., 2007; Zampieri et al., 2010).

More importantly, we found that the restricted three-dimensional trunk ROM during walking appeared early in the PD stage. Such deficits were impaired to a degree beyond the effects of aging, according to the comparison results with the age-matched control group, but they were not increased with the progression of the H-Y stages. A previous study showed that elderly participants increased voluntary control of their trunks in the coronal plane (medial-lateral direction) during walking (Hurt et al., 2010). Aging seems to have a greater impact on the mechanisms controlling walking stability in the coronal plane compared to young adults (Hurt et al., 2010). Greater trunk sway in the coronal direction was mainly associated with an increased risk of falls in both elder adults and patients with PD (Jehu and Nantel, 2018). As coronal trunk stability during walking is dependent on active sensory integration and this ability is impaired in PD (Siragy and Nantel, 2018), this might explain the more restricted coronal plane ROM in patients with PD than in age-matched controls in our study. Moreover, some studies reported that PD fallers had fewer movements for the trunk in both the anterior-posterior or vertical directions compared with PD non-fallers and the controls. A greater amount of trunk sway was reported in patients with PD during the medicated state than in the unmedicated state. Therefore, patients with PD displayed reduced walking ROM to compensate for trunk balance since the early stage, which may explain the compensatory strategies during the walking task.

The sagittal trunk angular velocity when standing and changing positions reduced progressively in patients with PD, and among them, the angular velocity during the stand-to-sit task was visible early in the PD stage and did not worsen with the H-Y stage. Previous studies showed contradictory results (Mak and Hui-Chan, 2002; Zampieri et al., 2010). The researcher suggested that sit-to-stand deficits appeared later than turning and walking deficits in PD, possibly related to weakness secondary to lack of mobility (Zampieri et al., 2010). However, our data suggested that stand-to-sit deficits might present early in PD. Accompanied by reduced walking trunk ROM, these variables might have high discriminative values for the early diagnosis of PD.

### The contribution of dopaminergic loss to PD imbalance progression

Loss of dopaminergic neurons is the main pathophysiology of PD. Dopamine reduction impairs the function of the basal ganglia, which results in cardinal symptoms. Previous studies demonstrated that the severity of the main PD motor symptoms, including rigidity and bradykinesia, were positively correlated with the striatal dopamine transporter (DAT) reduction; however, tremor was not (Kaasinen and Vahlberg, 2017; Kuribara et al., 2020). Therefore, it is speculated that not all PD symptoms can be completely explained by dopamine deficiency. Is the balance impairment related to striatal dopamine depletion? At present, there were inconsistent findings in the correlation between striatal DAT reduction and imbalance or instability of PD (Cabeleira et al., 2019; Corrêa et al., 2019). Notably, the effects of confounding factors, including age and white matter hyperintensities (WMHs), which were definitively factors correlated with balance and gait dysfunctions (Bohnen et al., 2011; Craig et al., 2019; Lee et al., 2020), were not adjusted in these studies (Cabeleira et al., 2019; Corrêa et al., 2019).

Before further analysis, we confirmed that DAT availability in the posterior putamen was well correlated with the MDS-UPDRS III score (Poewe et al., 2017; Lee et al., 2020). As indicated in previous studies that, while dynamic balance or stability was dependent on gait speed (Mirelman et al., 2019; Peterson et al., 2020; Wilson et al., 2020), gait speed, age, WMHs, and other confounding factors were adjusted in further regression analysis. Our results indicated that most balance parameters were not affected by decreasing dopaminergic uptake, and only the walking ROM in the coronal and transverse planes, two variables independent of disease progression, was significantly correlated with decreased DAT uptake in the striatum, mainly in the caudate. In PD, dopaminergic deficit in the striatum is a posterior-to-anterior gradient (Pasquini et al., 2019). The loss of motor automaticity is linked to the loss of dopaminergic innervation in the posterior striatum (Wu et al., 2015). The putamen receives information from the motor cortex and the premotor cortex. Dopamine neurons in putamen are mainly activated during walking (Ouchi et al., 2001). However, there was no correlation between parameters during walking with putamen dopaminergic uptake but with caudate. One recent study (Pasquini et al., 2019) showed that early caudate dysfunction in PD was not uncommon and that early caudate dopaminergic denervation increased the risk of gait impairments, while there has been no study on its association with balance in PD. Therefore, although our results showed the relationship between walking ROM and caudate DAT uptake, the specific pathophysiological mechanism remained unclear. Recent studies indicated that gait progression or balance control is not related to dopaminergic interventions (Pasquini et al., 2019; Zampogna et al., 2021), suggesting abnormal balance responses as a result of neurodegeneration in the non-dopaminergic extranigral pathways. Emerging evidence suggests that the gait and balance impairments in PD are the results of multisystem degeneration and deficits in other neurotransmission systems. The lateral vestibular system was also involved in postural and balance control (Karachi et al., 2010; Morris et al., 2019; Wilson et al., 2020). This might be the explanation for why most parameters of dynamic balance, especially those related to disease progression, are uncorrelated with DAT uptake. The cholinergic system dysfunction is often considered a significant component of PD gait and balance. A previous study concluded that cortical cholinergic denervation contributed more to gait and balance than nigrostriatal deficit alone (Bohnen et al., 2011). Attentional deficits following degeneration of brain cholinergic systems contribute to gait-balance deficits in PD, and a recent study indicated that target engagement of the nicotinic acetylcholine receptor (nAChR) stimulation improved gait-balance function in PD (Albin et al., 2021). Cholinergic neurons in the pedunculopontine nucleus were found to play a central role in controlling gait and balance (Grabli et al., 2013). However, phase 2 trials of cholinesterase inhibitors did not affect measures of dynamic balance in people with PD (Mancini et al., 2019). Therefore, the investigation of pathophysiological mechanisms responsible for dynamic balance impairment in PD remains rather limited. Further research is needed to define these mechanisms more clearly and to help choose the new target for imbalance.

Some limitations should be noted. First, the cognitive functions were involved in balance and gait. We assessed the MMSE and FAB scores. However, since we did not perform a more detailed neuropsychological assessment in these patients, we cannot exclude a possible relationship between mild cognitive functions and abnormal dynamic balance in PD. Second, there are some methods and many parameters to assess the dynamic balance, e.g., the harmonic ratio or Lyapunov exponents or detrended fluctuation analysis method (Bartsch et al., 2007; Hubble et al., 2015), but there are no recommendations for the most appropriate one (Siragy and Nantel, 2018). In our study, we used the wearable IMU system to quantify the TUG task to assess the dynamic balance. In the future, we could use other methods to verify the results. Third, concerning the potential effect of dopa-therapy on balance control, all measures were done in the “OFF” condition. However, our study did not further evaluate the effects of anti-parkinsonian medications on measures of balance and stability. The effectiveness of dopamine therapy on dynamic balance may help us understand the contribution of dopaminergic loss to balance in PD.

## Conclusion

Our study showed the evolution characteristics of dynamic balance in people with PD from early to advanced progression and the relationship between dopaminergic loss and instability progression. Patients with PD present with dynamic balance disorders in the early stages, and different parameters progress differently at distinct stages. Most parameters were not related to the striatum dopaminergic depletion, except for the positive correlation between ROM in the coronal and transverse planes and caudate DAT availability in the early stage. The broad lack of correlation between dopaminergic loss and dynamic balance impairment suggests that dopaminergic denervation contributes less to PD imbalance, and novel therapies targeting non-dopaminergic mechanisms should be further researched. In addition, early caudate dysfunction in the imbalance of PD should be of concern.

## Data availability statement

The raw data supporting the conclusions of this article will be made available by the authors, without undue reservation.

## Ethics statement

The studies involving human participants were reviewed and approved by the Research Ethics Committee of Xinhua Hospital, Affiliated to Shanghai Jiao Tong University School of Medicine. The patients/participants provided their written informed consent to participate in this study.

## Author contributions

Conceptualization of the study and supervision: ZL and YY. Project administration: JG, YW, JZ, LS, and NW. Funding acquisition: ZL, JG, and LS. Patient samples and data collection: JG, YW, JZ, and LS. Data analysis and data curation: XW, LS, and NW. Methodology: JG, XW, YW, and JZ. Writing the original draft: JG. Review and editing of the manuscript: JG, XW, YW, ZL, and YY. Support and assistance for the synthesis of radioactive markers: HW. All authors contributed to the article and approved the submitted version.
